# Corneal Ulcer in Dogs and Cats: Novel Clinical Application of Regenerative Therapy Using Subconjunctival Injection of Autologous Platelet-Rich Plasma

**DOI:** 10.3389/fvets.2021.641265

**Published:** 2021-03-18

**Authors:** Haithem A. Farghali, Naglaa A. AbdElKader, Huda O. AbuBakr, Eman S. Ramadan, Marwa S. Khattab, Noha Y. Salem, Ibrahim A. Emam

**Affiliations:** ^1^Department of Surgery, Anaesthesiology and Radiology, Faculty of Veterinary Medicine, Cairo University, Giza, Egypt; ^2^Department of Biochemistry and Molecular Biology, Faculty of Veterinary Medicine, Cairo University, Giza, Egypt; ^3^Department of Internal Medicine and Infectious Diseases, Faculty of Veterinary Medicine, Cairo University, Giza, Egypt; ^4^Department of Pathology, Faculty of Veterinary Medicine, Cairo University, Giza, Egypt

**Keywords:** corneal ulcer, autologous PRP, subconjunctival injections, dogs, cats, oxidative stress, MMPs

## Abstract

**Background:** Corneal ulcer could be a major source of distress in small animals, with many contributing agents. In recent years, few studies evaluated the efficacy of platelet-rich plasma (PRP) in healing corneal ulcers.

**Aim:** This study aimed to assess the ability of subconjunctival injection of autologous PRP in the treatment of corneal ulcers in dogs and cats as well as estimate the expression of matrix metalloproteinase (MMP)-2, MMP-9, and oxidative stress biomarkers in these patients.

**Methods:** A total number of 28 animals (16 cats and 12 dogs) were enrolled in this study. Each animal was subjected to clinical, neurologic, and ophthalmic examinations where the type of ulcer was documented. Tear samples were collected for evaluation of oxidative biomarkers and MMPs; conjunctival swabs were taken to identify the involved organism. PRP was prepared from each animal and given as subconjunctival injection; numbers of injections were done according to case response. Clinical follow-up was done and documented for each case.

**Results:** In cat patients, female and Persian cats were most affected; unilateral and superficial ulcers were most recorded. In male dogs, unilateral, and superficial ulcers were most recorded. FHV-1 was most identified in cats, while *Staphylococcus aureus* was most identified in dogs. Numbers of injections needed to achieve healing were recorded, with 50% of dogs needing two injections with 1-week intervals and 50% of cats needed three injections with 1-week intervals. Alterations in both oxidative biomarkers and MMPs were recorded in affected animals.

**Conclusion:** The use of autologous PRP as a subconjunctival injection in treating corneal ulcers in dogs and cats is effective. The number of injections is the case and corneal ulcer type-dependent.

**Clinical Significance:** Autologous PRP as a subconjunctival injection in treating corneal ulcer is a relatively cheap, safe method and can be done in the clinical setting.

## Introduction

Corneal ulceration is defined as a defect in the epithelium with stromal loss and/or inflammation (1). Corneal ulcers can cause a great deal of discomfort in patients, and it was accounted for up to 0.80% of conditions diagnosed in primary care practice in the UK (2). Contributing etiologies for this condition are numerous; trauma, bacterial or fungal infection, and immune-mediated diseases are the most reported causes (3).

In human medicine, extensive researches were conducted to treat corneal ulceration; recently, trials with platelet-rich plasma (PRP) were performed to assess its efficacy in healing corneal ulcers (4). These trials were conducted after promising findings were recorded in experimental animals (1, 5).

PRP, an autologous byproduct of blood that is very rich in platelets, earned wide recognition for its ability to heal various conditions (6, 7). As late as 1990, the term “regenerative medicine” was recognized (8). Platelets contain growth factors, cytokines, and integrins; these factors contribute to the proposed healing ability of PRP (9). The use of PRP is deemed convenient, cost-effective, non-immune provocation, and minimally invasive technique; the ability to administer it shortly after collection and preparation is an added merit (7). PRP can give essential components for the regeneration of tissue as scaffolds and growth factors (9).

PRP is rich in platelets, which are important for wound healing; they are rapidly deployed for injury sites, stick to it, and generate healing via releasing of numerous growth factors and cytokines (10). PRP was used successfully in treating dormant ulcers (11) and corneal epithelium defects following infectious keratitis (10). In human medicine, PRP was used extensively in the field of orthopedics, plastic, oral, and cardiovascular surgeries (11).

Matrix metalloproteinase (MMPs), a group of zinc-reliant extra-cellular endoproteinase, was postulated to play an integral role in corneal ulcer pathogenesis (12). MMP-2 and MMP-9 are thought to be the primary degrading enzymatic byproducts of corneal fibroblast and epithelial tissue (13, 14). MMPs catalyze basement membrane cleavage components (15). MMP-2 and MMP-9 were elevated in tears of patients with corneal diseases (16).

A strong refractive lens is supported by a combination of the corneal and precorneal tear film. In normal wear and tear mechanism, corneal extracellular matrix (ECM) is stabilized by a balance between collagen and ECM synthesis and their degradation by proteinases, which keep surveillance, remodel, and elimination of damaged corneal epithelial cells, and the abnormal component of ECM (15, 17, 18). These proteinases include MMPs, serine proteases, aspartic proteinases, and cysteine proteinases that exist in latent forms in normal conditions followed by activation during inflammation (13, 18–20). Both MMPs and serine proteinases play an important role in the normal and diseased corneal metabolism of human beings and animals (18, 21). MMPs play a vital role in all stages of wound healing and remodeling, and their overexpression results in excessive ECM degradation, which leads to tissue destruction and loss of visual function (22). MMPs are zinc-dependent enzymes that are classified according to their substrate into gelatinases, collagenases, stromelysins, and membrane-type MMPs (18). Gelatinases, such as MMP-2 and MMP-9, represent activity against collagen degradation products as well as against collagen types IV and V (15, 23). These proteases are mostly the predominant proteinases that are overexpressed in corneal ulceration in comparison with antiproteinases; tissue inhibitors of metalloproteinases (TIMPs) result in rapid degradation of collagen and other corneal ECM (15, 21).

Oxidative stress was described in numerous diseases in pet animal practice; both infectious and non-infectious etiologies were associated with damaging effects of oxidative stress mechanisms (24).

The state of elevated oxidant byproduct and reduction of antioxidant counterparts is known as oxidative stress (24). Malondialdehyde (MDA) is a lipid peroxidation byproduct that is usually measured to evaluate the oxidant arm in the body (25), while total antioxidant capacity (TAC) is used to crude estimate the status of the antioxidant system in the body (26).

This study aimed to assess the ability of subconjunctival injection of autologous PRP in the treatment of corneal ulcers in dogs and cats as well as estimate expression of MMP-2 and 9 and oxidative stress biomarkers in these patients.

## Materials and Methods

### Study Population, Clinical Examination, and Inclusion Criteria

This study was approved by the Animal Use and Care Committee at the Faculty of Veterinary Medicine, Cairo University, Egypt. A total number of 28 animals (16 cats and 12 dogs) were enrolled in this study. Animals were presented to the small animal clinics, faculty of veterinary medicine, Cairo University, and private clinics in the Cairo governorate. The corneal ulcer was documented at the time of admission, and verbal consent was given from animals' owners to participate in this study. Each animal was subjected to clinical examination, and signs were recorded at the time of admission.

The lack of previous medical/surgical interference of ulcer, at least 2 months of age, a clear definition of the morphological type of ulcer, and absence of other local or systemic illness were used as inclusion criteria in this study.

**Figure 1:**
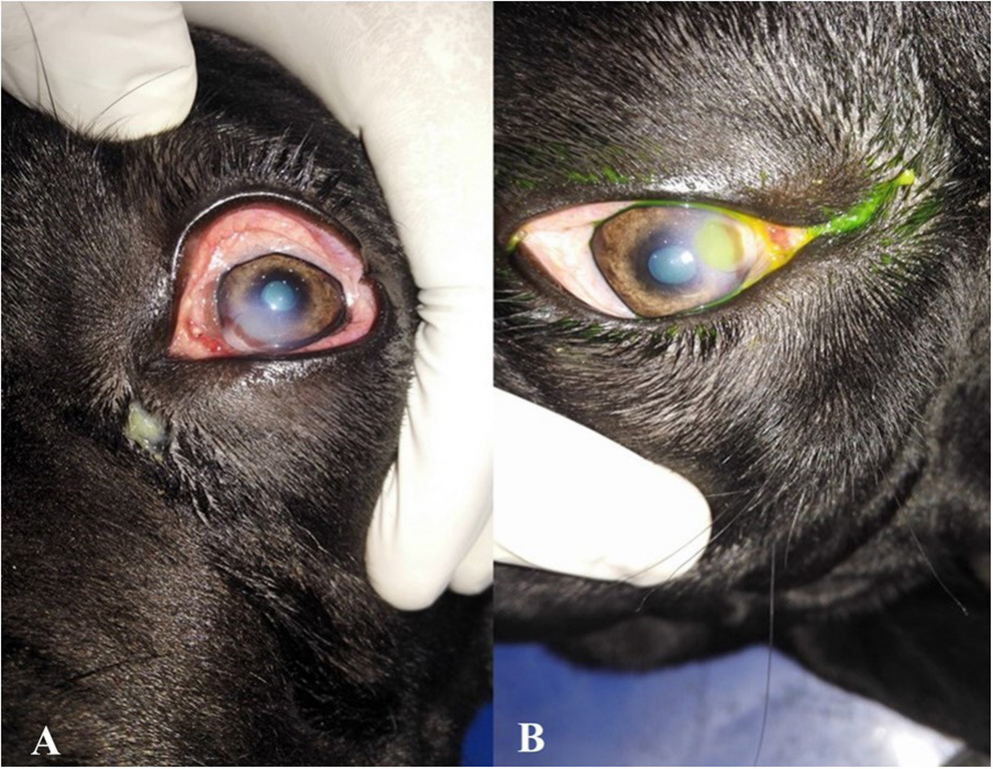
Photographs showing the clinical diagnosis of the ulcer by fluorescent dye. **(A)** Eye suffered from corneal ulcer, **(B)** after application of fluorescent dye showing type and depth of corneal ulcers.

Neurologic assessments of participants include menace response, palpebral, pupillary light, and dazzle reflexes. Ophthalmic examinations in a dark room with magnification and focal light source were performed. Ophthalmic examinations include direct ophthalmoscopy (Welch Allyn®, Skaneateles Falls, NY), the Schirmer tear test (Color Bar™ instrument; EagleVision, Inc., Memphis, TN), applanation tonometry (Tono-Pen® VET; Medtronic SOLAN, North Jacksonville, FL), and fluorescein dye (Fluorets® Chauvin, France) staining were done to suspected cases (Figure 1).

### Sampling and Laboratory Analysis

Tear fluid (diseased and treated) was collected from the inferior tear meniscus, causing the least irritation possible by the capillary tube method. The tear samples were then stored at −80°C until they were used for gelatin zymography (2).

Tear samples were taken by the method of the Schirmer test I. The sampled test strip was stored at −20°C (19).

Tear samples were used to estimate MDA, catalase (CAT), and TAC using specific test kits (Cat No. MD 25 29, CA 25 17, and TA 25 17, respectively; Bio-diagnostic, Egypt). The activity of MMP-2 and MMP-9 was detected in gelatin zymography by a method described by Hawkes et al. (27). Briefly, tear samples were separated by sodium dodecyl sulfate/polyacrylamide gel electrophoresis (SDS/PAGE) on 7.5% (w/v) gels, containing 1 mg/ml of gelatin under non-reducing conditions. Then, zymogram gels was washed twice for 15 min each in 2.5% (v/v) Triton X-100 and incubated overnight in development buffer (0.05 M of Tris/HCl, pH 8.8, 5 mM of CaCl_2_, 0.02% NaN_3_). Gels were stained with 0.1% Coomassie Brilliant Blue R250 in methanol:acetic acid:water (4.5:1:4.5, v/v/v). The zymogram gels were scanned in true color and then analyzed using commercially available software (myImageAnalysis Software; Thermo Scientific™) after conserving to grayscale.

### Culture and Molecular Biology

Tears samples and conjunctival swabs of all cases were streaked on mannitol salt agar and *Pseudomonas* agar base with CN supplement media.

Conjunctival swabs were attained by rotating a sterile cotton swab over the ventral conjunctiva and were deposited in a 2-ml tube containing sterile 0.9% NaCl solution. PCR was conducted to detect *Chlamydophila felis* and FHV-1 according to methods described before (28, 29); primers and expected amplicons are tabulated in Table 1.

**Table 1:** Primer sequences for *Chlamydophila felis* and FHV-1.

**Organism**	**Primer**	**Expected amplicon**	**References**
C. felis	Oligo 420 (CAG GAC ATC TTG TCT GGC TTT AA) Oligo 423 (CGG ATG CTG ATA GCA TCA CAC CAA GT)	277 bp	(28)
FHV-1	FHV-tkf (GTT GTC GGT GGT ATC TAT GC) FHV-tkr (GAG GTT CTC GTG GAA GTG TT)	306 bp	(29)

### Preparation of the Platelet-Rich Plasma and Injection

PRP was prepared using a double-spin method as a protocol previously described by Kecec et al. (30). Briefly, blood from each animal was collected on 3.8% sodium citrate solution, soft spin at 250 × g/10 min was applied, the top and middle layers were then collected, and hard spin was performed at 2,000 × g/10 min followed by removal of platelet-poor plasma and activation of PRP by 20 mM of CaCl_2_ and incubation at 37°C/1 h. Centrifugation was then applied at 3,000 × g/20 min for recovering activated PRP.

### Identification of Ulcer Types, Treatment Plans, and Complications

For superficial ulcers, a loss of part of the epithelium was the base of categorization. Deep ulcers that spread into/through the stroma and might cause severe scarring; fluorescein stain was taken up by exposed corneal stroma and with green appearance. Fluorescein stain defined the corneal ulcer margin and revealed further details of the surrounding epithelium. Fluorescein dye test was applied in all the cases and used to identify the different sites of the corneal ulcer and their size.

After the ulcer type was identified, two treatment options were done: (1) subconjunctival injection of PRP and, in case of entropion, (2) surgical correction of entropion in affected cases followed by subconjunctival injection of PRP. Surgical correction was done under general anesthesia, as follows: atropine sulfate (1% at 0.05–0.1 mg/kg b.wt.; Adwia Co., S.A.E, Egypt) and xylazine (Xyla-Ject 12% at 1 mg/kg b.wt.; Adwia Co., S.A.E, Egypt) were used as pre-medication, followed by ketamine at 10–20 mg/kg b.wt. (Sigma-Tec, Egypt) for induction and maintenance (31).

Surgical correction of entropion was done following the Hotz–Celsus procedure. Briefly, the removal of a crescent-shaped section of skin from the entropic region of the eyelid was made via a 6400 Beaver blade. At first, a parallel skin incision was done to the rolled-in portion of the eyelid at the eyelid margin by 2–3 mm; a second skin incision was bent away from the eye and commenced at one end of the first incision and end up at the other. The crescent-shaped section of the skin was removed with tenotomy scissors. The surgical gap was sutured via staples, where the first suture joined the epicenter of the first and second incisions. The second and third sutures were placed bilaterally to the first suture. The lingering defects were sutured every 3–4 mm till seamless juxtaposition of skin margins (32).

Subconjunctival injection of autologous PRP was used in each case, and bilaterally if the bilateral ulcer was identified at the time of admission.

**Figure 2:**
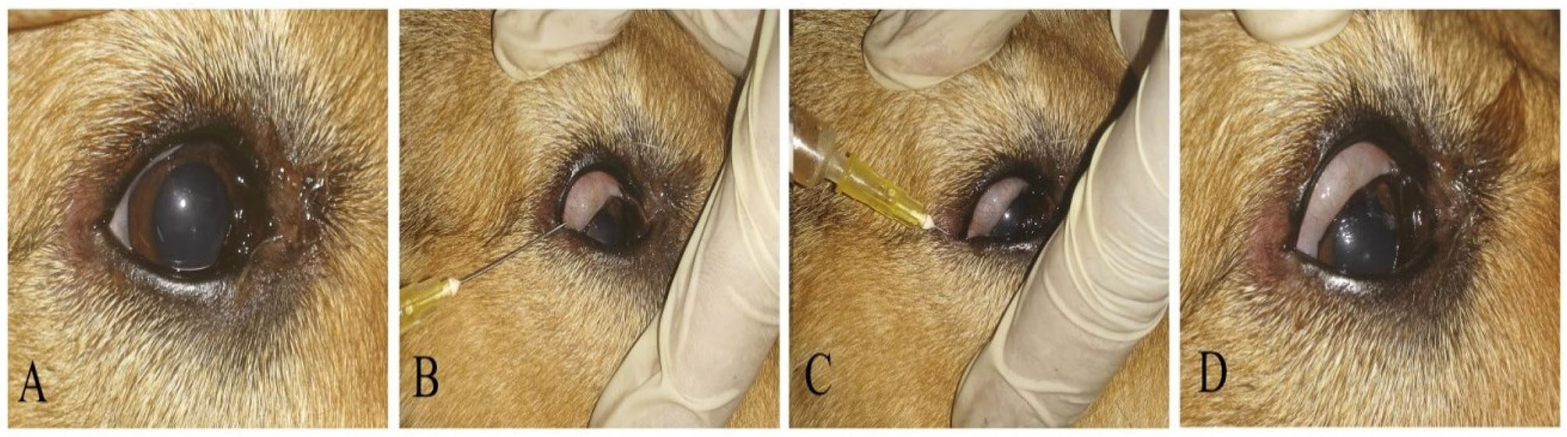
A photography series showing the technique of PRP conjunctival injection in the upper and lower eyelids and conjunctival pouch. **(A)** Before injection, **(B,C)** PRP injections in upper and lower eyelids and conjunctival pouch, **(D)** eye after injection.

Subconjunctival injection of autologous PRP was scheduled and timed for each case; the injection was applied under complete aseptic conditions and repeated till a complete curative clinical response with 1-week interval (Figure 2). Clinical evaluation was conducted via digital photographs of the ulcer at the baseline and at each time of subconjunctival injection (Table 2).

**Table 2:** Patient signalments and description.

**Case no**.	**Breed**	**Age (M)**	**Sex**	**Ulcer description**	**Clinical appearance**	**Suspected cause**
**Cat**						
1	Himalayan	3	Male	Unilateral corneal sequestrum	Black plaque developed in the paracentral feline cornea with superficial vascularization. Chemosis and severe hyperemia were recorded	Trauma (scratch)
2	Persian	6	Female	Bilateral mid-stromal corneal ulcers	Visible divot or defect in stroma, usually more pronounced and extensive corneal edema, fluorescein-positive, white-yellow cellular infiltrate may be visible, anterior uveitis (miosis, refractory to pharmacologic mydriasis, aqueous flare)	Infection
3	Mixed	18	Male	Unilateral superficial corneal ulcer	Break in epithelium only, fluorescein positive, focal corneal edema, no divot, or defect in corneal stroma	Trauma (scratch)
4	Persian	2	Male	Bilateral mid-stromal corneal ulcers	Visible divot or defect in stroma, usually more pronounced and extensive corneal edema, fluorescein-positive, white-yellow cellular infiltrate may be visible, anterior uveitis (miosis, refractory to pharmacologic mydriasis, aqueous flare)	Infection
5	Mixed	20	Female	Unilateral corneal sequestrum	A brownish plaque was visualized in the central feline cornea (coagulation necrosis of the corneal stroma) with superficial vascularization	Trauma (scratch)
6	Mixed	3	Female	Bilateral mid-stromal corneal ulcers	Visible divot or defect in stroma, usually more pronounced and extensive corneal edema, fluorescein-positive, white-yellow cellular infiltrate may be visible, anterior uveitis (miosis, refractory to pharmacologic mydriasis, aqueous flare)	Infection
7	Mixed	8	Female	Unilateral superficial corneal ulcer	Break in epithelium only, fluorescein positive, focal corneal edema, no divot, or defect in corneal stroma	Trauma (scratch)
8	Persian	9	Female	Unilateral superficial corneal ulcer	Break in epithelium only, fluorescein positive, focal corneal edema, no divot, or defect in corneal stroma	Trauma (scratch)
9	Mixed	15	Male	Unilateral superficial corneal ulcer	Break in epithelium only, fluorescein positive, focal corneal edema, no divot, or defect in corneal stroma	Trauma (scratch)
10	Persian	36	Female	Unilateral superficial corneal ulcer	Break in epithelium only, fluorescein positive, focal corneal edema, no divot, or defect in corneal stroma	Trauma (scratch)
11	Siamese	12	Female	Unilateral corneal sequestrum	Circumscribed black plaque developed in the center of the affected cornea with superficial vascularization	Trauma (scratch)
12	Persian	7	Male	Bilateral superficial corneal ulcer	Break in epithelium only, fluorescein positive, focal corneal edema, no divot, or defect in corneal stroma	Trauma (scratch)
13	Persian	3	Female	Bilateral mid-stromal corneal ulcers	Visible divot or defect in stroma, usually more pronounced and extensive corneal edema, fluorescein-positive, white-yellow cellular infiltrate may be visible, anterior uveitis (miosis, refractory to pharmacologic mydriasis, aqueous flare)	Infection
14	Persian	18	Female	Unilateral superficial corneal ulcer	Break in epithelium only, fluorescein positive, focal corneal edema, no divot, or defect in corneal stroma	Trauma
15	Siamese	28	Male	Unilateral superficial corneal ulcer	Break in epithelium only, fluorescein positive, focal corneal edema, no divot, or defect in corneal stroma	Trauma (scratch)
16	Siamese	9	Female	Unilateral superficial corneal ulcer	Break in epithelium only, fluorescein positive, focal corneal edema, no divot, or defect in corneal stroma	Trauma (scratch)
**Dogs**						
17	Rottweiler	36	Male	Unilateral		Entropion
18	Pit-bull	18	Male	Unilateral superficial corneal ulcer	Break in epithelium only, fluorescein positive, focal corneal edema, no divot, or defect in corneal stroma	Trauma
19	Rottweiler	12	Male	Unilateral superficial corneal ulcer	Break in epithelium only, fluorescein positive, focal corneal edema, no divot, or defect in corneal stroma	Trauma
20	Pekinese	96	Male	Unilateral keratoconjunctivitis sicca		Trauma
21	Siberian Husky	48	Male	Unilateral superficial corneal ulcer	Break in epithelium only, fluorescein positive, focal corneal edema, no divot, or defect in corneal stroma	Trauma
22	Saint Bernard	36	Male	Unilateral melting corneal ulcers	Severe corneal edema, variable degrees of ocular pain, pronounced swelling, and malacia of the cornea resulting in a drooping or bulging appearance. Melting may commonly manifest in dogs as “drilled out” defects in the stroma rather than stromal swelling and bulging. Cellular infiltrate may or may not be apparent. Usually considerable anterior uveitis (aqueous flare, miosis, hypopyon)	Infection
23	Griffon	48	Male	Unilateral superficial corneal ulcer	Break in epithelium only, fluorescein positive, focal corneal edema, no divot, or defect in corneal stroma	Trauma
24	German Shepherd	12	Male	Unilateral		*Chemical burn*
25	Griffon	16	Female	Unilateral superficial corneal ulcer	Break in epithelium only, fluorescein positive, focal corneal edema, no divot, or defect in corneal stroma	Trauma
26	Saint Bernard	24	Male	Bilateral		Entropion
27	Yorkshire	168	Female	Unilateral superficial corneal ulcer	Break in epithelium only, fluorescein positive, focal corneal edema, no divot, or defect in corneal stroma	Trauma
28	Rottweiler	72	Female	Unilateral superficial corneal ulcer	Break in epithelium only, fluorescein positive, focal corneal edema, no divot, or defect in corneal stroma	Trauma

### Statistical Analysis

Data were compared using the *T*-test, with *p* ≤ 0.05 considered significant. Data are represented as mean ± SE, using SPSS for Windows, Version 16.0. Chicago, SPSS Inc. (released 2007).

## Results

Data of each animal, description of the lesion, and the number of injections needed are presented in Table 2. In cat patients, females were more likely to be affected than males (62.5 and 37.5%, respectively). Persian cats were more presented compared with mixed-breed and Siamese cats (50, 31.2, and 18.7% respectively). In dog patients, male dogs were predominant than female (75 and 25%, respectively), Rottweiler, Saint Bernard, and Griffon breeds were overpresented in this study.

The ulcer morphological type for cats and dogs is shown in Table 3. The superficial corneal ulcer was more predominant in cat patients, followed by deep, corneal sequestration and a mid-stromal corneal ulcer (43.7, 25, 18.75, and 12.5%, respectively). For dogs, superficial corneal ulcer (58.3%) was more predominant followed by mid-stromal ulcer (25%). Unilateral corneal ulcer was prominent in cat patients than bilateral (87.75 and 12.5%, respectively). The unilateral ulcer was most common in dog patients than bilateral (90 and 10%, respectively). Data showed that unilateral corneal ulcer was common in dogs and cats, with similar frequency.

**Table 3:** Corneal ulcer distribution and types in dogs and cats.

**Types of corneal ulcer**	**Dogs**			**Cats**			**Total**
	**Unilateral**	**Bilateral**	**Total**	**Unilateral**	**Bilateral**	**Total**	
Superficial	6	1	7	5	2	7	14
Mid-stromal	3	–	3	2	–	2	5
Deep (descemetocele)	1	–	1	4	–	4	5
Melting	1	–	1	–	–	–	1
Corneal sequestrum	–	–	–	3	–	3	3
Total	11	1	12	14	2	16	28

Subconjunctival injection needed for each animal is shown in Table 4. In dog patients, 50% (6/12) of cases needed two injections (1-week interval), while in cats, 50% (8/16) needed three injections.

**Table 4:** Number of injections needed for each case.

**Type of corneal ulcer**		**Dogs**					**Cats**					**Total**
		**Number of injections**				**Total**	**Number of injections**				**Total**	
		**Once**	**Twice**	**Three times**	**Four times**		**Once**	**Twice**	**Three times**	**Four times**		
Superficial	Unilateral	–	4	2	–	6	1	1	2	1	5	11
	Bilateral	–	–	1	–	1	–	1	1	–	2	3
Mid–stromal	Unilateral	–	2	1	–	3	–	1	1	–	2	5
Deep (descemetocele)	Unilateral	–	–	1	–	1	–	–	3	1	4	5
Melting	Unilateral	–	–	–	1	1	–	–	–	–	–	1
Corneal sequestrum	Unilateral	–	–	–	–	–	–	–	1	2	3	3
Total		–	6	5	1	12	1	3	8	4	16	28

Suspected causes of ulcers are listed in Table 2. In cat patients, trauma was the leading possible cause at the time of admission in 75% of cases (12/16). In dog patients, trauma was the possible cause at the time of admission in 66.6% of cases.

Results of culture and molecular identification are shown in Table 5. In cat patients, FHV-1 was most the predominant organism followed by *C. felis*. In dogs, *Staphylococcus* was the most predominant organism.

**Table 5:** Microorganisms involved in corneal ulcers in dog and cat patients.

**Case no**.	**Breed**	***Feline herpes virus type−1 (FHV-1)***	***Chlamydophila felis***	***Staphylococcus aureus***	**Pseudomonas *sp*.**
1	Himalayan	+ve	+ve		
2	Persian	+ve			
3	Mixed	+ve		+ve	
4	Persian		+ve		
5	Mixed	+ve			
6	Mixed		+ve		
7	Mixed	–ve	–ve	–ve	–ve
8	Persian	–ve	–ve	–ve	–ve
9	Mixed	+ve			
10	Persian	+ve			
11	Siamese	+ve			+ve
12	Persian		+ve		
13	Persian	+ve			
14	Persian	+ve			
15	Siamese	+ve			
16	Siamese	+ve			
**Dogs**					
17	Rottweiler	–ve	–ve	–ve	–ve
18	Pit-bull			+ve	
19	Rottweiler				+ve
20	Pekinese				+ve
21	Siberian Husky			+ve	
22	Saint Bernard			+ve	
23	Griffon			+ve	
24	German Shepherd			–ve	–ve
25	Griffon			–ve	–ve
26	Saint Bernard			+ve	
27	Yorkshire			–ve	–ve
28	Rottweiler			+ve	

**Figure 3:**
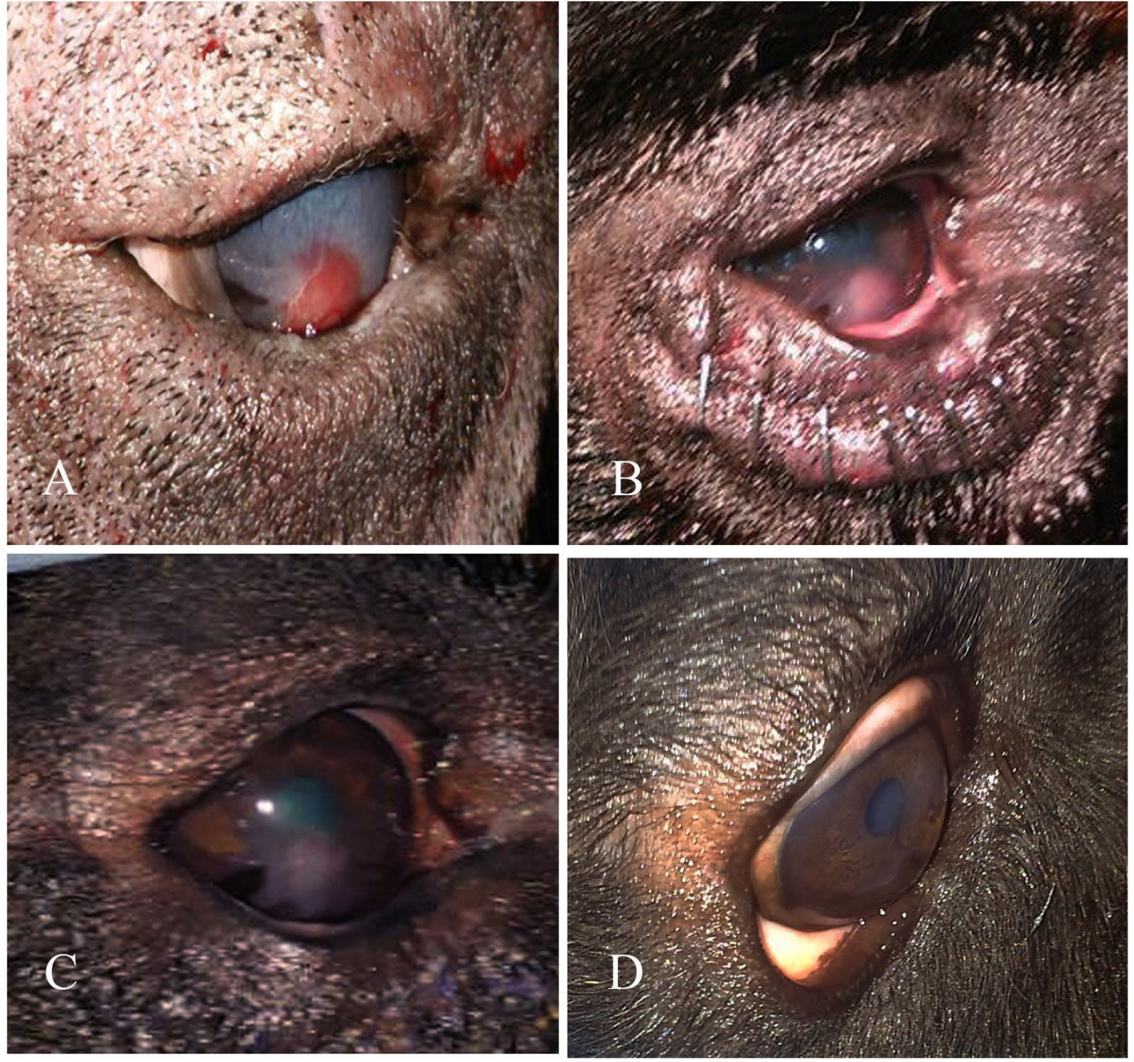
A photography series of Rottweiler dog showing **(A)** entropion with corneal ulcer **(B)** post-operative after surgical correction of entropion and injection of PRP **(C)** reduction of corneal ulcer size after 1-week post-injection **(D)** complete healing of ulcer after 2 weeks of injection.

**Figure 4:**
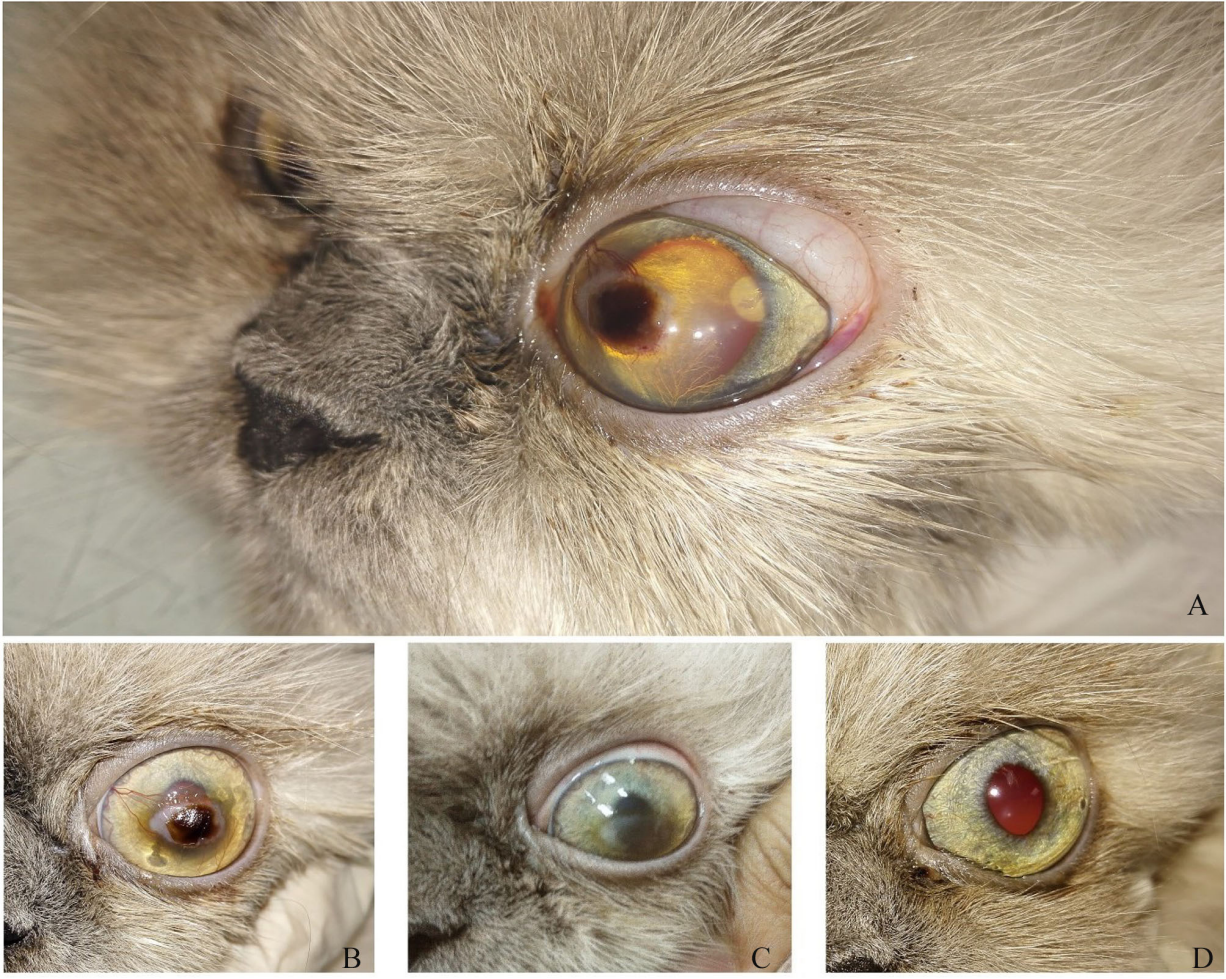
A photography series of Himalayan cat showing **(A)** superficial ulcer with sequestration **(B,C)** reduction of corneal ulcer size after 1,2-month post-injection, respectively, **(D)** complete healing of ulcer after 3 months of injection.

**Figure 5:**
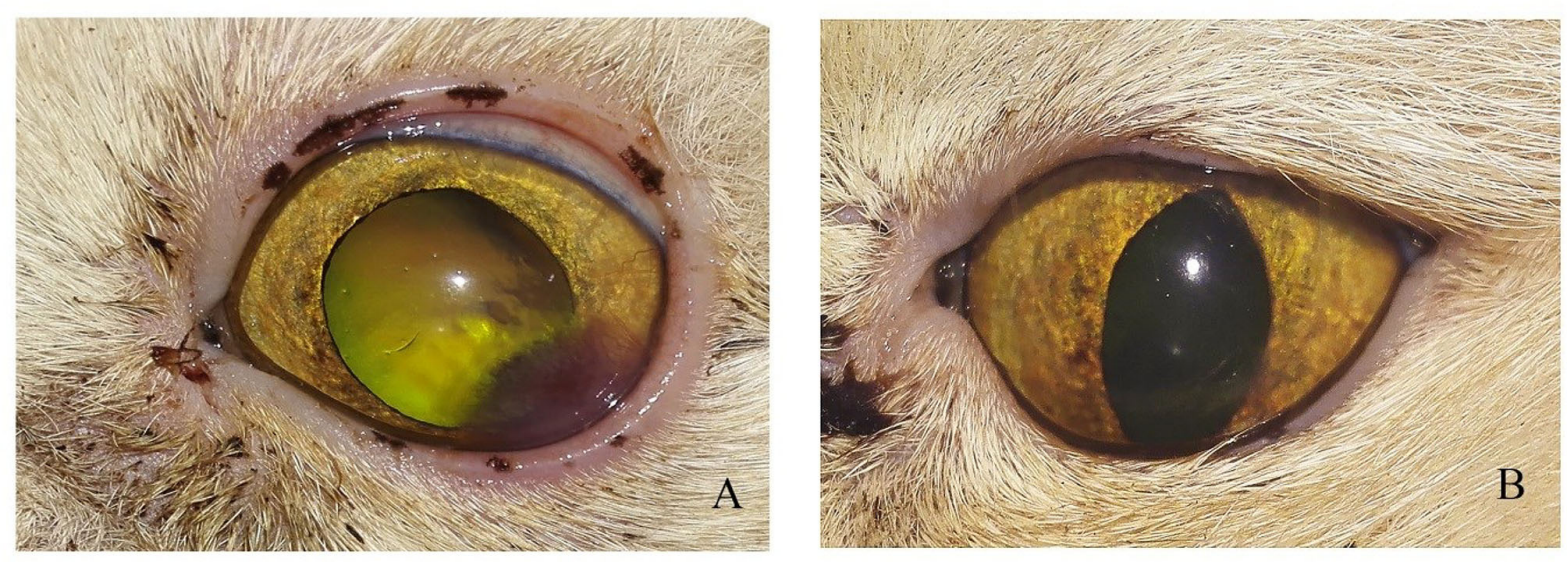
Photographs of a short hair cut showing **(A)** Stromal ulcer **(B)** complete healing of ulcer after 1 month of injection.

**Figure 6:**
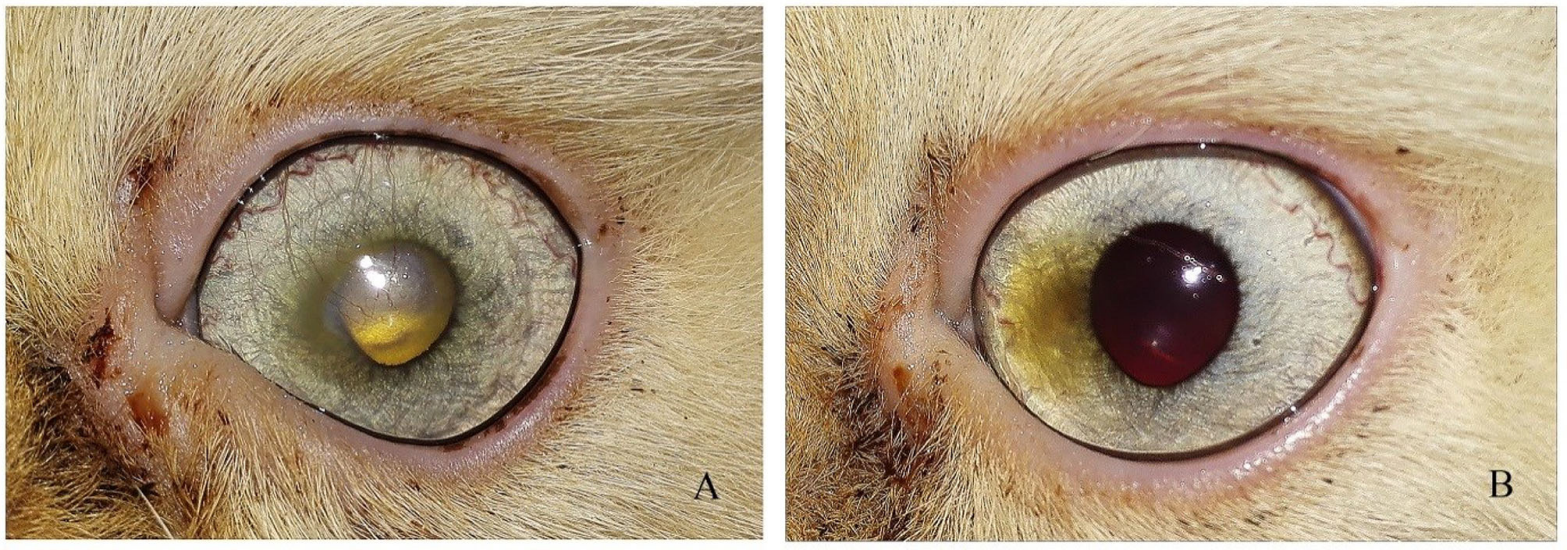
Photograph series of a short hair cut showing **(A)** keratitis **(B)** complete healing of ulcer after 1 month of injection.

**Figure 7:**
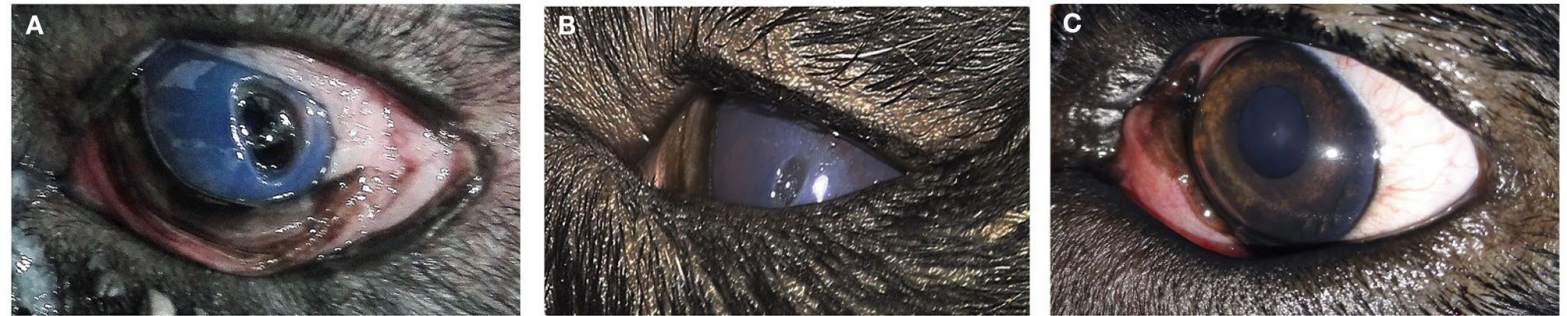
A photography series of Rottweiler dog showing **(A)** deep ulcer with keratitis **(B)** reduction of ulcer size after 2-week post-injection **(C)** complete healing of ulcer after 3 months of injection.

**Figure 8:**
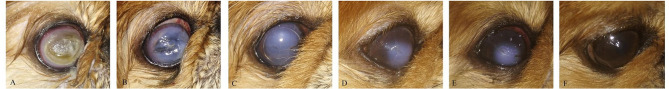
A photography series of Pekingese dog showing **(A)** melting ulceractive keratitis **(B–F)** gradual healing and reduction of keratitis after 2-week post-injection till complete healing at 3 months.

**Figure 9:**
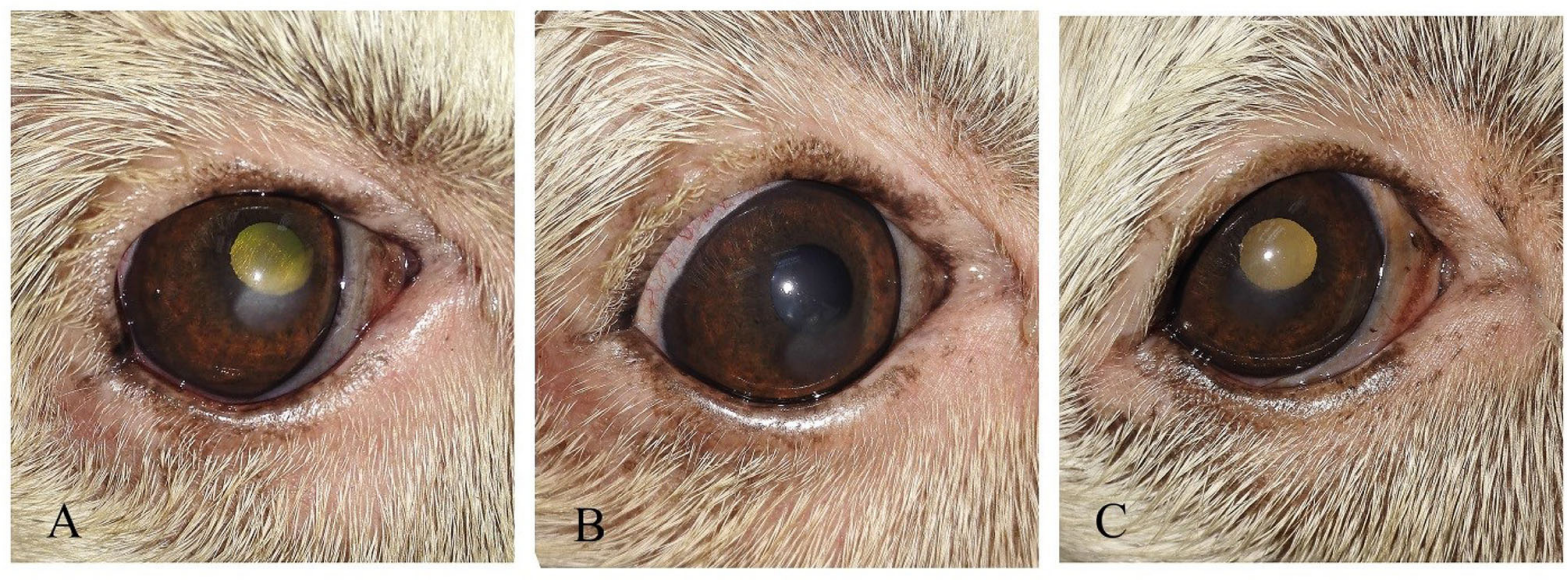
**(A)** photography series of Griffon dog suffering from keratitis **(B)** reduction of ulcer size after 1-week post-injection **(C)** complete healing of ulcer after 2 weeks of injection.

Clinical description and follow-up are shown in Figures 3–9, Table 2. In the gross examination, the majority of the cases showed mild-to-moderate congestion, lacrimation, and slight corneal opacity. All the reported cases responded to menace reflex and direct pupillary light reflex. There was continuous pawing, itching, irritation, pain, and photophobia in ulcerative cornea, which lead to excessive lacrimation.

In cats, the majority of cases with superficial unilateral and mid-stromal ulcers needed two injections of PRP till complete healing, the deep (descemetocele) type needed three injections, and the melting type needed four injections, with ~50% of cases receiving three injections. In dogs, two injections of PRP are required for healing in 50% of cases. Some cases needed four injections as in the corneal sequestrum type. The size of ulcer reduced in first 2 weeks in the superficial type and complete healing at 2–4 weeks, while in deep ulcer, reduction of size occurs at 2–4 weeks, and complete healing and transparency of the cornea were maintained at 2 months and reached till 3 months in corneal sequestrum (Table 4).

**Figure 10:**
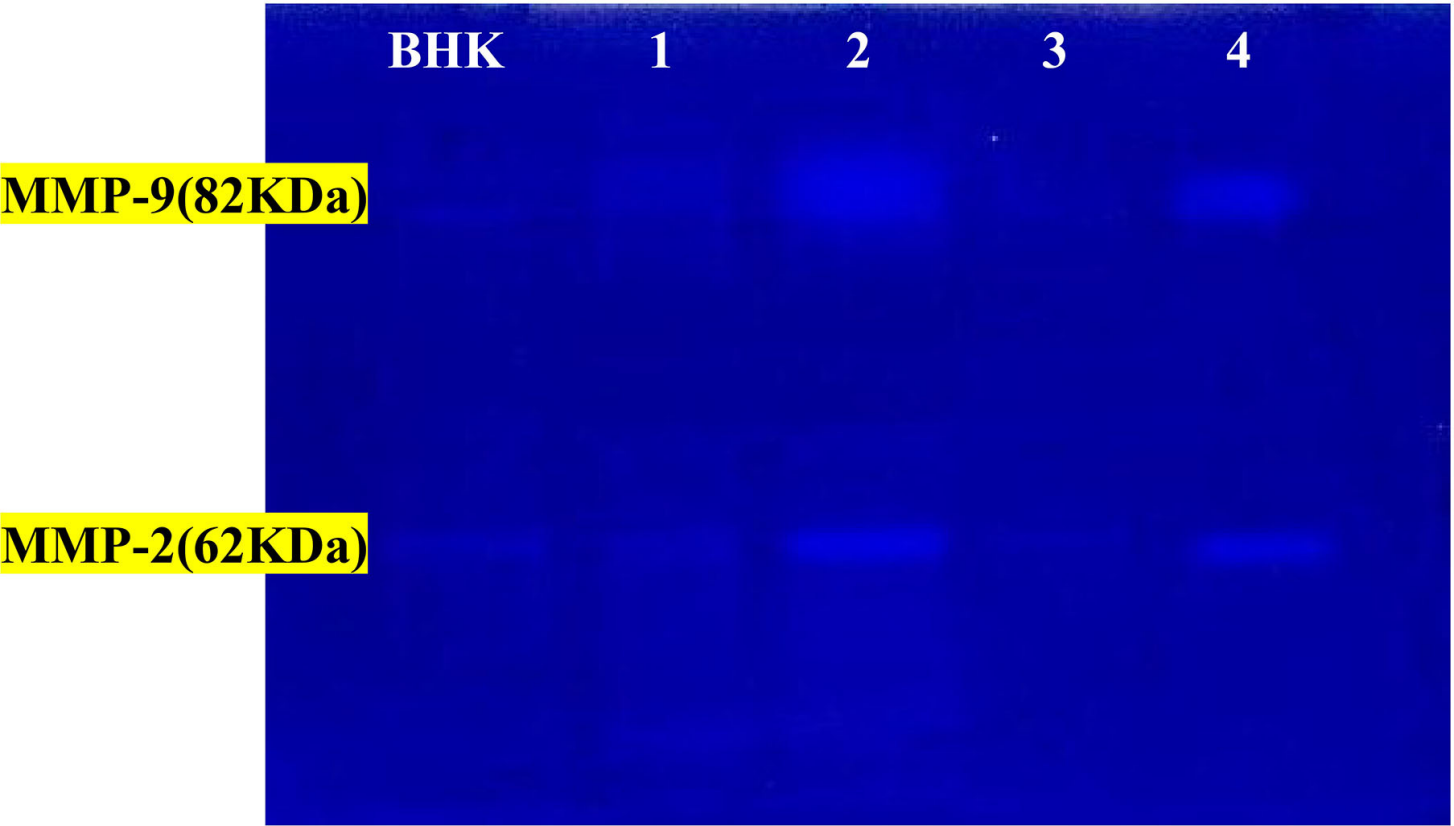
The activity of MMP-2 and MMP-9 by gelatin zymography. Lanes 1 = PRP-group; lanes 2 = control +ve in cat; lanes 3 = PRP groups; lanes 4 = control +ve group in dog. BHK lane is a control marker from baby hamster kidney cells transfected with active MMP-9 (82 kDa) that are indicated by arrows and MMP-2 (62 kDa).

Results of oxidative biomarkers and MMPs are shown in Tables 6–8, Figure 10. In cat patients, a significant increase in MDA associated with a significant decrease in CAT and a non-significant decrease in TAC was reported in tear samples of cats with corneal ulcer as compared with treated cats, while dogs showed a significant decrease in both CAT and TAC associated with the significant increase in MDA in tear samples of affected dogs compared with treated dogs.

**Table 6:** Oxidative stress biomarkers in dog corneal ulcer tear sample.

**Parameter/unit**	**PRP**	**Control +ve (diseased)**
TAC	1.10 ± 0.08	0.96 ± 0.51
MDA	1.33 ± 0.10	3.79 ± 0.77^*^
Catalase	521.83 ± 25.43	369.29 ± 20.00^*^

Data are represented as mean ± SE;

* *p ≤ 0.05 considered significant; SPSS program version 16.00*.

*PRP, platelet-rich plasma; TAC, total antioxidant capacity; MDA, malondialdehyde*.

**Table 7:** Oxidative stress biomarkers in cat corneal ulcer tear sample.

**Parameter/unit**	**PRP**	**Control +ve (diseased)**
TAC	0.97 ± 0.07	0.43 ± 0.04^*^
MDA	1.87 ± 0.20	4.21 ± 0.62^*^
Catalase	529.14 ± 28.41	280.96 ± 22.64^*^

Data are represented as mean ± SE;

* *p ≤ 0.05 considered significant, SPSS program version 16.00*.

*PRP, platelet-rich plasma; TAC, total antioxidant capacity; MDA, malondialdehyde*.

**Table 8:** Gelatin zymography findings.

**MMPs**	**Cat**		**Dog**	
	**PRP**	**Control +ve** **(diseased)**	**PRP**	**Control +ve** **(diseased)**
MMP-2%	3.1 ± 0.1	5.6 ± 0.3^*^	3.1 ± 0.1	5.4 ± 0.1^*^
MMP-9%	4.3 ± 0.2	13.3 ± 0.4^*^	4.1 ± 0.1	7.6 ± 0.2^*^

Data are represented as mean ± SE;

* *p ≤ 0.05 considered significant; SPSS program version 16.00*.

*PRP, platelet-rich plasma; MMP, matrix metalloproteinase*.

The activity percentages of both MMPs (MMP-2 and MMP-9) were significantly high in the corneal ulcer control positive group (diseased group), which significantly decreased (*p* ≤ 0.05) after treatment with autologous PRP treated groups in both animals.

## Discussion

### Results and Key Findings

Corneal ulceration is defined as a defect in the epithelium with stromal loss and/or inflammation (1). The conventional treatment of corneal ulcer with different types of antibiotics (chloramphenicol, gentamicin sulfate, neomycin sulfate, tobramycin, ciprofloxacin, gatifloxacin, and ofloxacin) was sometimes associated with harmful effects on corneal re-epithelialization during healing (33).

This study examined the effect of subconjunctival injection of autologous PRP in different types of corneal ulcers in dogs and cats. The main findings of this study are that autologous PRP heals different types of corneal ulcers in both dogs and cats and the number of injections needed is case-dependent; however, most of the dogs needed two injections for complete healing, while the cat counterpart needed three injections. The most common microorganism implicated in corneal ulcers in cats is FHV-1, while in dogs, *Staphylococcus*. Corneal ulcers in dogs and cats are associated with oxidative process and alterations in MMPs.

The secondary finding of this study is that superficial corneal ulcer is the more predominant type in dogs and cats. Female Persian cats appeared to be most affected, while male dogs were overpresented compared with females.

### Findings and Critics

#### Patient Data

Female Persian cats were overpresented, with corneal sequestration mainly seen in Siamese, Persian, and Himalayan in this study. All breeds, gender, and age group are susceptible to developing corneal ulcers (34, 35), though owners' preference to raise female cats may be implicated. In this study, male dogs were more presented than female. Rottweiler, Saint Bernard, and Griffon dogs were most likely to be affected; these findings contradict those reported by O'Neill et al. (2), as they reported that Boxer and Pug were most likely to be affected. The difference in breed presentation could be related to breeding preference and popularity.

#### Ulcer Morphological Type

In cat patients, superficial ulcer followed by corneal sequestration was the most predominant morphological type. The superficial ulcer is associated with defective epithelium with the baring of stroma, while descemetocele is usually associated with the absence of stroma at the ulcer base (34). The revival of latent FHV-1 is linked to conjunctivitis with superficial ulceration as a sequel, which may be unilateral or bilateral (36). Corneal sequestration usually occurs secondary to ulcerative keratitis of chronic nature (37). In dogs, superficial corneal ulcer with unilateral involvement was the most common morphological type. Other reports showed that unilateral ulcer was overrepresented (38). In the study conducted by Kim et al. (39), they found that superficial corneal ulcer represented 44% of cases followed by a deep ulcer that entails about 2/3 of the stroma.

#### Etiological Agents

FHV-1 was implicated in most of the cat patients in this study. It is postulated that many cases of corneal ulcer in cats originated mainly from FHV-1 infection (40–43). Other causes of ulceration are trauma, entropion, and chemical trauma (34). In dogs, *Staphylococcus aureus* was isolated from 50% of patients; in one report performed on 19 dogs with corneal ulcers, *Staphylococcus* was the most prominent isolate (38). A canine corneal ulcer usually arises from canine herpes virus or spontaneous chronic ulcer as primary causes with entropion, trauma, and corneal degeneration as secondary causes (28, 44–48).

#### Subconjunctival Injection of Autologous Platelet-Rich Plasma

PRP, especially E-PRP, was advocated for usage in human medicine for its cost-effectiveness, ability to heal the corneal wound as sole/adjuvant therapy, and relative safety (49); it was used successfully in the treatment of dormant corneal ulcer and was proven to help reduce inflammation and pain in these patients (11). The rationale for deploying such methods was described extensively in the literature, with the absence of clotting factors and the presence of many growth factors as the main advocate for its usage (49). In one human study, PRP was used as eye drops, the healing was improved significantly, and complete healing was possible in most of the participant cases (11).

The eyes' position in the body makes them continuously exposed to numerous agents and traumatic events (50). These circumstances make them react to any insult constrained and may lead to vision loss (51). In recent years, PRP was used in the regeneration and reconstruction of tissues, plastic, and cardiovascular surgeries as well as it aids in corneal lesion healing (1). A subconjunctival route was chosen to administer PRP in both dogs and cats. In two reports dealing with rabbits and dogs, the subconjunctival injection was used instead of drops and can be given as a single shot contrary to the drop form (5, 52).

PRP is a powerful, effective, and safe cure for various wound-healing processes. In this study, half of the dog patients required two injections at 1 week apart, regardless of the morphological type, while approximately half of the cat patients required three injections at 1 week apart regardless of the morphological type of ulcer. Compared with subconjunctival injection, PRP drops were used in clinical practice twice daily for 15 days in dogs after defrosting PRP every time it was used (53).

In one study conducted in rabbits, PRP contains growth factors that were directly applied on the injured surface of the cornea and in turn modulate repair and reduce formation of scars (50). As PRP is not categorized as a drug agent by the Food and Drug Administration (FDA), a protocol of administration is yet to be defined (54). PRP administration improves the recovery of muscle and tendon injuries (55). Cytokines and growth factors within platelets initiate and boost tissue healing via stimulation of cell proliferation, migration, and angiogenesis (6). In a human study dealt with a non-healing ulcer, PRP drops were employed, and they concluded that it is an efficient and cost-effective way to treat neurotrophic keratitis (56). PRP rushes healing and enhances epithelium (57). PRP is inexpensive, easy to obtain, and possesses the merit of being a non-surgical alternative with lesser side effects (1).

#### Clinical and Photographic Follow-Up

The utilization of PRP as a subconjunctival injection is a recent approach in human and veterinary medicine. In the superficial type, the ulcer size is reduced in the first 2 weeks, and complete healing occurs at 2–4 weeks. In deep ulcer, reduction of size occurs at 2–4 weeks with complete healing, and transparency of the cornea was maintained at 2 months and till 3 months in cases of the corneal sequestrum. Conventional treatment usually employs antibiotics; for instance, galifloxacin 0.3% might heal the cornea within 15 days; however, 78% of indolent ulcers could be healed with the combination of chondroitin and tobramycin in up to 4 weeks (33). The expected action of PRP based on the extraordinary concentration of epithelia-trophic growth factors might lead to faster healing (53). In the study conducted by Ferrone Neto et al. (33), they concluded that the addition of PRP to conventional therapy improved clinical cure.

#### Corneal Ulcer and Oxidative Stress

When a balance between reactive oxygen species (ROS) production and the ability of an antioxidant to remove them is disrupted, the oxidative process will ensue. Cellular damage and peroxidation of lipid in the cellular membrane are expected (58, 59). In the present study, corneal ulcers in both dogs and cats showed oxidative stress in the form of elevated MDA and reduction in TAC and CAT in tear samples, which normalized to normal levels after therapy. Abundance in ROS overwhelms the antioxidant system, and the eye tissue became overloaded with ROS (60). The increase in MDA level could be attributed to increasing ROS output or as compensation for the reduction in antioxidants (24). Alterations in the hydration of the cornea are factored in resultant oxidative stress (61). MDA elevation is a good indicator for lipid peroxidation (27). In ulcerative keratitis, the elevation in MDA and reduction in CAT and superoxide dismutase (SOD) were recorded and attributed to inflammation (62).

The enzymatic antioxidant as CAT plays a role in protecting ocular tissue; CAT is known to convert H_2_O_2_ to water; both CAT and SOD act together as frontrunners in antioxidant effort to remove ROS (63, 64). TAC showed a significant reduction in corneal ulcers regardless of their morphological status. TAC represents a collective act of all antioxidants, and it could give a crude assessment of antioxidant levels in the body (25). As the frontrunners act as a first-line defense in the antioxidant system (65), a reduction in TAC is expected. TAC could be a simple tool to assess antioxidant systems regardless of system antioxidant modus operandi (66). Experimental keratitis was associated with oxidative stress in the cornea (62). Oxidative stress in corneal affections is linked to many factors, and this process can disrupt the cornea, which ranged from a reduction in visual perception to vision loss (58).

#### Corneal Ulcer and Matrix Metalloproteinases

MMPs are expressed in low levels in normal non-injured corneal tissues, as MMP-2 exists in an inactive form and MMP-9 is undetectable with induction and activated or upregulated in response to cytokines and growth factors (13, 18). MMP-9 is incorporated in the early stages of corneal epithelial wound healing, while MMP-2 is associated with the remodeling in the later stages of corneal wound healing (67). Gelatin zymography is a method used for easy detection of latent and active MMPs. Because the active forms of MMPs are water-soluble enzymes, it is difficult to detect by immunohistochemical or molecular biological method (68).

In the present study, the activity of gelatinases (MMP-2 and MMP-9) was significantly high in the corneal ulcer control positive group, which significantly decreased after treatment with autologous PRP in both dogs and cats. These results agreed with those in (21, 69), which stated that MMPs and serine proteinases were predominant in horse and dog corneal ulceration that released into the microenvironment of injured corneal tissue from leukocyte infiltration. The precorneal tear film serine proteinase levels were significantly higher in dogs with indolent ulcers vs. normal controls (70).

Singh et al. (68) demonstrated that the active forms of MMP-9 are present in tears of severe ulcerative and ocular surface disorder patients with no detection in the control group. Also, an injury without bacterial infection resulted in an increase in both MMP-2 and−9 and a slight but significant downregulation of TIMP-1 in mouse corneal ulcer (19). In the administration of anti-interleukin (IL)-1 antibodies before infection, it showed a significant decrease in MMP levels and a significant change in the time course of TIMP induction. This reduction in MMP-9 may contribute to reduced corneal damage.

There is evidence that in ulcerative corneal disease, alteration of the ratio of MMPs to TIMPs plays a role in progressive stromal degradation (71). Furthermore, the expression of collagenolytic and gelatinolytic MMPs is increased in bacterial corneal ulcers of mice and rabbits (19). The levels of MMP-9 and MMP-2 were found to be significantly elevated in climatic droplet keratopathy patients (72). These data suggest that MMPs produced by resident corneal cells and polymorphonuclear (PMN) leukocytes may play a role in early epithelial keratitis and the ulcerative process in the late phase after corneal HSV-1 infection in BALB/c mice (73). Mulholland et al. (74) showed that MMP-2 and−9 were overexpressed in anterior keratectomy (AK) and lamellar keratectomy (LK) wounds in rabbits and then rapidly fell to low levels after epithelial closure.

MMP-2 is produced by corneal epithelial cells, stromal keratocytes/fibroblasts, and PMN leukocytes and acts as housekeeping proteinase in the normal cornea by degrading occasionally damaged collagen fibers (75, 76). However, MMP-9 is secreted by corneal epithelial and stromal cells; it is responsible for destroying the adhesive structure of the epithelial cell basement membrane before overt stromal ulceration and delays the re-epithelialization of the ulcerated cornea (15, 18).

During the corneal injury, wound healing is started by recruitment of leukocytes, fibroblasts, and vascular endothelial cells to begin healing phases, including inflammation, angiogenesis, re-epithelialization, granulation tissue formation, and ECM deposition in response to MMPs and other proteinases (18). In severely damaged corneas, corneal stromal ulceration propagates through epithelial basement membrane degeneration because of overexpression of MMPs, elevated plasmin activity from inflammatory cells, and pro-inflammatory cytokines such as tumor necrotic factor-alpha and transforming growth factor-beta (12, 69, 77).

In the case of corneal infection, these proteinases are oversecreted by infectious organisms that simultaneously enhance corneal damage (19, 21, 78). In context, *Pseudomonas* produces two types of MMPs, alkaline and elastase proteases, that are responsible for aggressive ulcerative keratitis associated with this microbe (77). Also, bacterial and fungal infections induce recruitment of corneal epithelial cells, corneal stromal fibroblasts, and PMN leukocytes in the tear film that upregulate cytokines (IL-1, IL-6, and IL-8), leukocyte infiltration, and angiogenic factors that induce explosive production of MMPs to lead to stromal scarring and loss of corneal transparency (12, 18, 19, 78).

The treatment for severe ocular surface disorders has been a long-standing challenge and has been advocated by a variety of medical techniques and surgical procedures. As a result of the elevation of MMP levels, tear film parallels the severity of the corneal disease. These levels diminish when treatment is initiated and as the ulcer heals (69). Thus, PRP rich with their growth factors has been recommended for the treatment of corneal ulcer and ocular surface disorders to reduce the progression of stromal ulcer and to minimize corneal scarring through reduction of tear film proteolytic activity (68). Normalizing proteolytic activity in the tear film is an objective sign of effectiveness in the treatment of corneal ulcers and ocular surface disorder.

#### Comparison With Human Literatures

In this study, PRP was useful in treating corneal ulcers in small animal patients. It was used in many human studies for treating different types of ulcers (10, 56, 79–81). This study optioned for PRP usage as subconjunctival injection that could be administered weekly. In human patients, it was given mostly as eye drops (56, 79). A unique way developed by Alio et al. (80) combined E-PRP clot with fibrin membrane for corneal perforation closure. PRP was used as a solid application in ulcer in another report (81). It appears that subconjunctival injection of PRP in human patient is not widely used as compared with eye drops. This study opted to utilize subconjunctival administration instead of eye drops; this method, in the authors' opinion, albeit gives incredible results in human patients (82), has many hindrances for pet patients, as the need for repeated daily administration of product is the biggest hindrance. In one case report that dealt with refractory corneal ulcer, the patient was advised to administer PRP every 2 h for 15 days (79). As owner's compliance could not be ensured, subconjunctival method seems more appropriate for small animal patients.

In this study, most of dog patients required at least two injections with 1-week interval, while cats needed at least three injections with 1-week injection. In human literature, an average of three to eight drop application/day was reported (4, 82–85); the follow-up differs according to case, 2 months in dry eye diseases (11) and 6 months in recurrent corneal erosion (86).

The cause of ulcer in this study was often multifactorial, with infectious agent involvement in some cases. PRP was able to heal different types of corneal ulcers. In human literatures, PRP was used to cure ulcers with various etiologies (11, 80, 83, 87). PRP was efficiently used in treating persistent corneal abnormalities following an infectious condition (10).

This study lacked a control group to act as comparison arm. In human literature, there was a split between studies that deployed comparison arm (88–90); other reports did not deploy a comparison arm (11, 84, 91, 92). In a recent systematic review conducted by You et al. (93), they analyzed the results of 35 clinical studies that dealt with different ocular diseases and utilized platelet products as line of therapy, under corneal ulcer in their table description. They reported 16 studies that deployed PRP in treating different corneal ulcers: six studies used comparison arms and 10 studies did not utilize/report involvement of comparison group.

### Limitations and Strength

This study dealt with a novel way of treating different types of corneal ulcers in dogs and cats. The study uses PRP, a cheap and applicable method, as the main therapy. However, there was no comparison group using the conventional way of treatment to compare its efficacy and ability to improve healing. Comparing the results of subconjunctival injection of PRP with those of traditional therapy should be investigated in both dogs and cats in further studies.

## Conclusion

Subconjunctival injection of autologous PRP is cost-effective, easy to apply, and improves the healing of corneal ulceration in both dogs and cats regardless of their morphological type. The number of injections needed to reach complete healing is case-dependent rather than morphological type-dependent. A corneal ulcer is associated with local oxidative stress manifested by elevation in MDA and reduction in TAC and CAT; this oxidative process is present independent of the morphological type. MMP-2 and MMP-9 increased with corneal injury, though they normalize after PRP administration.

## Data Availability Statement

The original contributions presented in the study are included in the article/supplementary material, further inquiries can be directed to the corresponding author/s.

## Ethics Statement

The animal study was reviewed and approved by the Institutional Animal Care and Use Committee, Cairo University (Vet CU 20022020127). Written informed consent for participation was not obtained from the owners because the owners were informed orally about the steps of the study and all contact data of the owners are available in the archive of the small animal clinic, faculty of veterinary medicine, Cairo University, and participated private clinics in Cairo governorate.

## Author Contributions

HF established the research hypothesis and designed the study. HF, NA, and IE collected the data and applied the subconjunctival PRP injections. HA, ER, and NS prepared the PRP and performed the biochemical laboratory analysis. MK prepared the PRP and performed the statistical analysis. All authors participated in writing and approved the final manuscript.

## Conflict of Interest

The authors declare that the research was conducted in the absence of any commercial or financial relationships that could be construed as a potential conflict of interest.
